# Measuring the biomechanical properties of cell-derived fibronectin fibrils

**DOI:** 10.1007/s10237-024-01918-3

**Published:** 2024-12-26

**Authors:** Caleb J. Dalton, Soma Dhakal, Christopher A. Lemmon

**Affiliations:** 1 Department of Pharmaceutics, Virginia Commonwealth University, 410 N. 12th St., Richmond, VA 23298 USA; 2 Department of Chemistry, Virginia Commonwealth University, 1001 W. Main St., Richmond, VA 23284 USA; 3 Department of Biomedical Engineering, Virginia Commonwealth University, 401 W. Main St., Richmond, VA 23284 USA

**Keywords:** Fibronectin, Optical tweezers, Elastic modulus, Nonlinear elasticity, Viscoelasticity, Fibrils, Extracellular matrix, Mechanobiology, Biomechanics

## Abstract

Embryonic development, wound healing, and organogenesis all require assembly of the extracellular matrix protein fibronectin (FN) into insoluble, viscoelastic fibrils. FN fibrils mediate cell migration, force generation, angiogenic sprouting, and collagen deposition. While the critical role of FN fibrils has long been appreciated, we still have an extremely poor understanding of their mechanical properties and how these mechanical properties facilitate cellular responses. Here, we demonstrate the development of a system to probe the mechanics of cell-derived FN fibrils and present quantified mechanical properties of these fibrils. We demonstrate that: fibril elasticity can be classified into three phenotypes: linearly elastic, strain-hardening, or nonlinear with a “toe” region; fibrils exhibit pre-conditioning, with nonlinear “toe” fibrils becoming more linear with repeated stretch and strain-hardened fibrils becoming less linear with repeated stretch; fibrils exhibit an average elastic modulus of roughly 8 MPa; and fibrils exhibit a time-dependent viscoelastic behavior, exhibiting a transition from a stress relaxation response to an inverse stress relaxation response. These findings have a potentially significant impact on our understanding of cellular mechanical responses in fibrotic diseases and embryonic development, where FN fibrils play a major role.

**Supplementary Information:**

The online version contains supplementary material available at 10.1007/s10237-024-01918-3.

## Introduction

Fibronectin (FN) is a 250 kDa glycoprotein that is present in a soluble form at high concentration in the blood plasma (Mao and Schwarzbauer 2005). Cells bind to soluble plasma fibronectin via transmembrane integrins, which transmit contractile forces of the actomyosin cytoskeleton via focal adhesions to FN (Zhong et al. 1998). Stretching FN exposes buried cryptic FN-FN binding sites, which leads to the incorporation of additional soluble plasma FN molecules (reviewed in Dalton and Lemmon (2021)). This in turn leads to assembly of networks of insoluble fibrils in an iterative process known as fibrillogenesis. FN fibrils are the primordial matrix assembled by fibroblasts during wound healing and embryogenesis. In embryogenesis, FN fibrils are essential for some of the earliest developmental steps: in Xenopus embryos, gastrulation fails in the absence of FN, and the embryos have significant cardiovascular defects (Marsden and DeSimone 2001). In wound healing, the initial fibrin clot binds to factor XIII which in turn facilitates binding with FN (Clark et al. 2003). FN fibrils serve both as a means of structurally stabilizing the clot (Grinnel 1980) and as a network that facilitates cell migration into the wound to direct immune response (Clark et al. 1982).

Fibronectin fibrils exhibit unique mechanical properties. Previous work has shown that FN fibrils are extremely extensible and can be stretched up to four times their resting length (Ohashi et al. 1999), which distinguishes them from other extracellular matrix (ECM) polymers such as collagen and laminin. FN fibril extensibility is of particular interest with regards to the field of mechanobiology. Studies have shown that cells sense and respond to the mechanical properties of the surrounding tissue (Muncie and Weaver 2018; Chen 2008; Engler et al. 2006); given that FN fibrils serve as a link between the cell and surrounding tissue, it is impossible to fully understand the cellular mechanoresponse without understanding the biophysics of fibrils.

FN fibril formation requires stretch to open and expose cryptic domains that facilitate FN-FN binding (Zhong et al. 1998). Because of this, FN does not easily polymerize in cell-free conditions as other polymerizable proteins do, such as actin (Doolittle et al. 2013), collagen (Kreger et al. 2010), or tubulin (Sept 2007). Several groups have devised methods of assembling FN fibrils in cell-free conditions (Little et al. 2008; Feinberg and Parker 2010; Salmerón-Sánchez et al. 2011; Klotzsch et al. 2009). However, these methods do not replicate the morphology or extensibility of native cell-derived fibrils (discussed in Dalton and Lemmon (2021)). This is most likely in part due to different pulling speeds: cells assemble fibrils on the order of hours to days, whereas cell-free fibrils are assembled on the order of seconds.

We and others have previously utilized microfabricated pillar arrays (MPAs), which are arrays of micron scale cantilever beams comprised of polydimethyl siloxane (PDMS) (Lemmon et al. 2005; Tan et al. 2003). MPAs are prepared so that only the free (top) end of the pillar is adhesive for cells; traditionally, these have been used to measure cell contractile forces by quantifying the deflections of the pillars (Lemmon et al. 2005; Tan et al. 2003; Han et al. 2012; Chen et al. 2004); however, we have previously demonstrated that MPAs also serve as an ideal substrate for FN fibril deposition (Scott et al. 2015; Lemmon et al. 2009). As cells attach, they stretch FN on the top surface of the pillars and use this as an initiation site of FN fibril growth. These fibrils are almost entirely planar in the top plane of the pillars and are thus suspended away from the bottom surface. Since the spacing between micropillars and their geometry are highly tunable, MPAs create a unique environment to drive FN fibril size and interrogate fibril mechanics.

Given the substantial extensibility of FN fibrils and the role of FN fibrils in mediating mechanical signals between cell and surrounding tissue, understanding the mechanics of FN fibrils is extremely relevant. While several studies have investigated the mechanical properties of individual FN domains (Abu-Lail et al. 2006) and/or cell-free self-assembled FN fibers (Klotzsch et al. 2009; Smith et al. 2007; Szymanski et al. 2017), none of these studies have utilized cell-derived, native FN fibrils. While information can be gleaned from self-assembled FN fibers, these often differ dramatically in organization, size, and extensibility (reviewed in Dalton and Lemmon (2021)). As a point of example, Klotzsch et al. measured the stress–strain relationship of self-assembled FN fibers using a MEMS force sensor (Klotzsch et al. 2009); the forces needed to strain these fibers were on the order of 30 µN, and fibers had diameters of roughly 30 $\mu m$, resulting in stresses on the order of 2 Pa at strains of 3–4%. This is in stark contrast to the cell-derived fibrils in this study, which required forces on the order of hundreds of pN to strain, had diameters over 30X smaller, and exhibited stresses nearly 5 orders of magnitude higher at strains of 3–4%.

Optical tweezers have proven to be a useful tool in characterizing the function of large-scale protein structures. For example, optical tweezers-based studies of motor proteins such as RNA polymerase, dynein, kinesin, and myosin have revealed information that is otherwise not possible and thus revolutionized our understanding of how these proteins work (Capitanio and Pavone 2013; Fazal et al. 2016). These studies have shown that mechanical manipulation of biomolecules using optical tweezers allows quantitative determination of kinetic and thermodynamic parameters that help us understand how these systems work in cells. While optical tweezers have been used to study the binding strength of FN to fibroblasts (Thoumine et al. 2000) and to bacteria (Simpson et al. 2002), the technique has not been used to study the mechanics of FN fibrils.

**Fig. 1:**
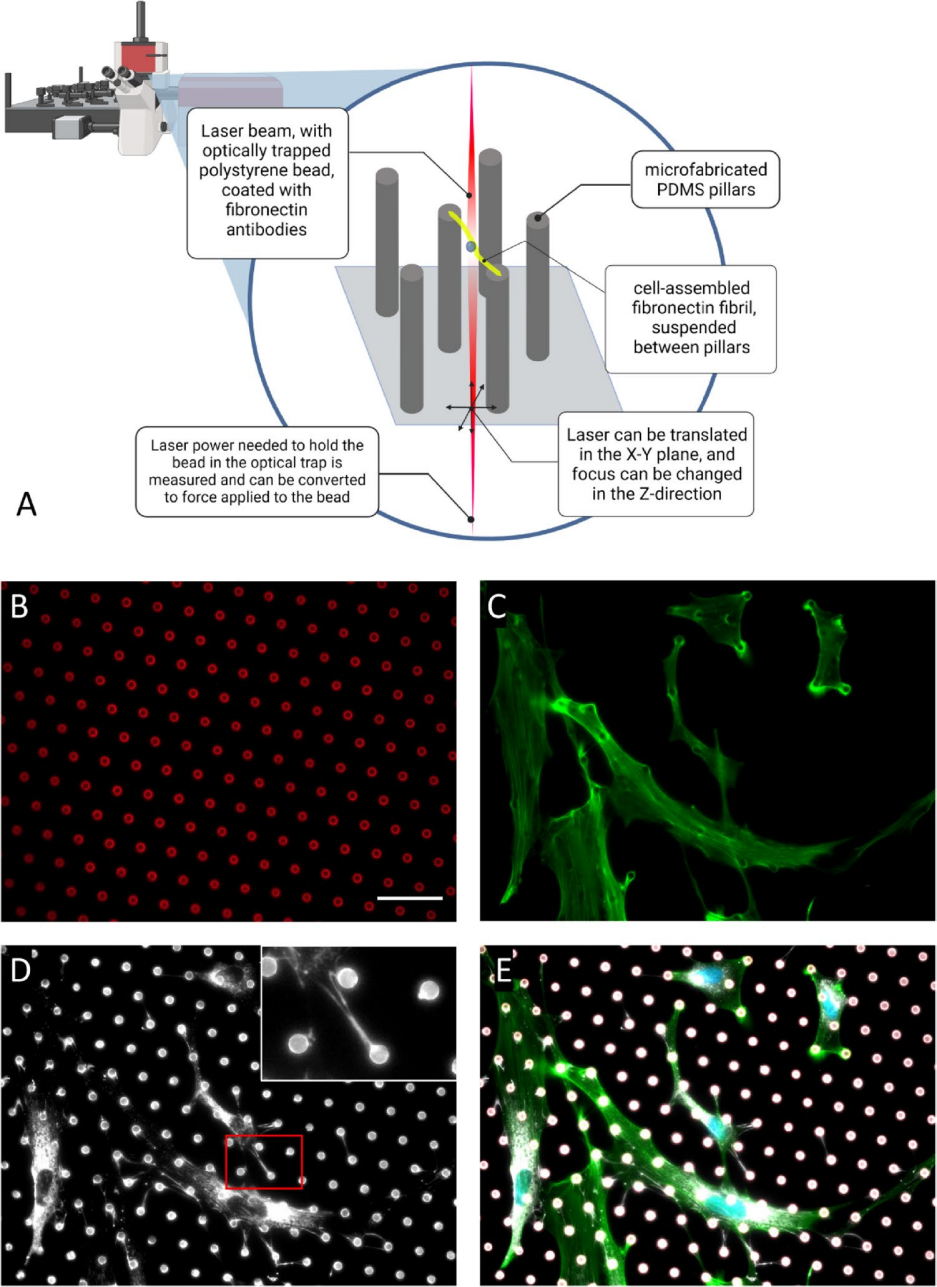
Optical tweezing of cell-derived fibronectin fibrils. **A** A schematic of the experimental system. Cells assemble FN fibrils onto the top surface of microfabricated pillars and are subsequently removed. Remaining FN fibrils are stretched with optical tweezers to measure stress–strain relationships. **B**–**E** Immunofluorescence images of **B** the microfabricated pillars, **C** actin, **D** fibronectin, and **E** the composite image. Cell-derived fibrils are highlighted in the red box. Scale bar is 20 microns

In the current work, we present an assay in which we combine both the microfabricated pillar array and optical tweezer technologies (Fig. 1A) to quantify FN fibril mechanics. Cell-derived FN fibrils were assembled by human adipose-derived mesenchymal stem cells (Fig. 1B–E). Cells were subsequently removed, leaving only FN fibrils suspended between neighboring MPA pillars. These fibrils were then pulled via FN antibody-coated polystyrene beads trapped in an optical tweezer. We demonstrate that the fibrils are primarily comprised of FN, lack significant decoration by other ECM proteins, and can be repeatedly and reproducibly stretched. Finally, we report mechanical properties of cell-derived FN fibrils, including characterization of fibril elastic behavior and quantification of viscous properties that change with repeated pulling, suggesting a mechanical memory of FN fibrils.

## Results

### Cell-derived assembly of FN fibrils that span micropillars

**Fig. 2:**
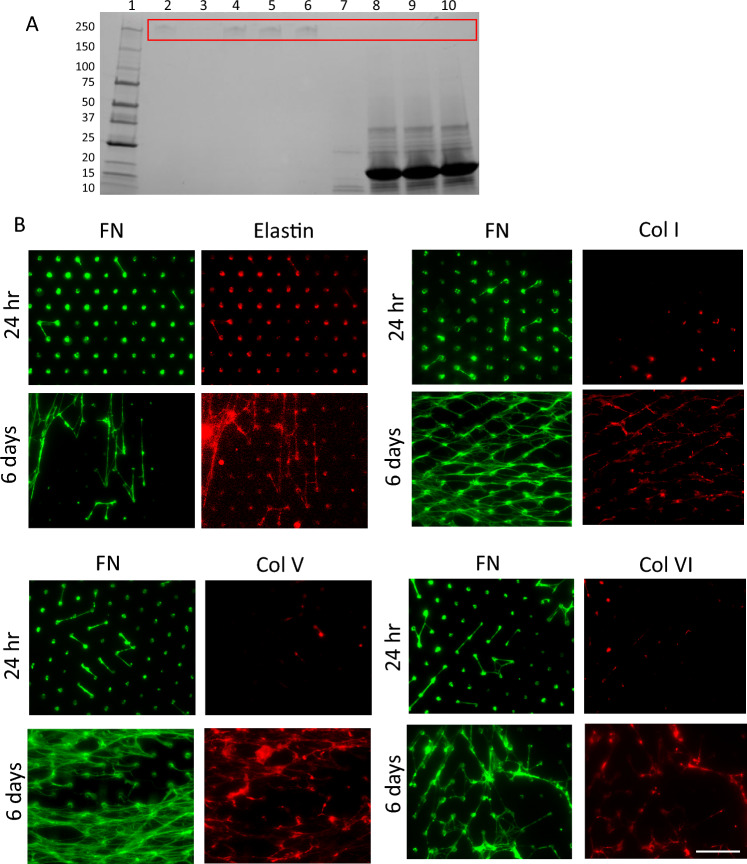
Characterizing the composition of cell-derived FN fibrils. **A** To ensure that FN is not cleaved by the cell extraction buffer, FN was incubated with increasing time exposed to the buffer, and no cleavage was detected (lanes 4–6). As a control, FN was incubated with trypsin exposed for the same times (lanes 8–10), where all FN is cleaved. **B** Dual labeling of FN and common ECM proteins to determine if ECM proteins are colocalizing with FN fibrils. Scale bar = 20 microns

In order to measure the biomechanical properties of cell-derived FN fibrils, we first needed to create a platform that encouraged the production of intact, cell-derived, suspended fibrils that can be used to probe the mechanics that are representative of in vivo fibrils. Prior work from our group has demonstrated that cells are capable of assembling FN fibrils that span the tops of neighboring micropillars (Lemmon et al. 2009; Scott et al. 2015). As a first step in the current work, we investigated whether cells could be removed from the micropillar assays such that intact FN fibrils were left behind. Figure 1B–E shows representative images of ASC52telo human adipose-derived mesenchymal stem cells plated on MPAs. Following cell removal, only FN fibrils remain. To ensure that the cell extraction buffer does not cleave FN, we incubated soluble FN with the extraction buffer over a range of concentrations and times (Fig. 2A). As a control, FN was incubated with trypsin, which is known to cleave FN. Results suggest that for all times and concentrations investigated, FN is not cleaved by the extraction buffer, as indicated by the 250 kDa band observed on the gel. This is in contrast to incubation with trypsin, which fully cleaved FN into fragments.

Another concern of the assay is the composition of the fibrils that remain after cell extraction. Many ECM proteins bind to FN fibrils, including members of the Collagen family, fibrillins, elastins, and fibulins (reviewed in Dalton and Lemmon (2021)). ASC52telo cells exhibit significant FN secretion and fibrillogenesis with little laminin or Collagen I deposition after a 2-week seeding period (Novoseletskaya et al. 2020) but also deposit Collagen V and Collagen VI (which bind FN) within 48 h (Hoefner et al. 2020). While fully characterizing the composition of FN fibrils is difficult given the insolubility of assembled fibrils, we investigated the colocalization of several ECM proteins that are known to conjugate with FN fibrils (Fig. 2B). Immunofluorescence imaging detected colocalization of Elastin, Collagen I, Collagen V, and Collagen VI with FN at 6 days of fibril growth but was less visible at 24 h, suggesting that binding of these ECM proteins occurs later (Elastin can be seen deposited on the tops of MPA pillars at 24 h but is not visibly colocalized with FN fibrils). While these studies are qualitative, they suggest that there is a change over time in the degree of proteins attached to the fibrils. In the current work, we focused exclusively on probing the mechanics of fibrils assembled for 24 h in order to minimize the potentially variable influence of other ECM proteins. It is worth noting that the decoration of FN fibrils with other ECM proteins at later time points is a potential strength of the assay: future studies will investigate how fibril mechanics change over time, including how additional ECM protein incorporation alters mechanics. The current assay would allow for a comparison between the mechanics of an “older” fibril, including its decoration with other ECM components, versus a “younger” fibril with ECM components added exogenously. This would allow for distinct measurements of the effects of time and ECM protein decoration on FN fibril mechanics. These types of studies are not possible in assays that use cell-free FN fibers derived solely from stretched soluble FN (such as those conducted in Klotzsch et al. (2009)).

### Distribution of fibril sizes and geometries

**Fig. 3:**
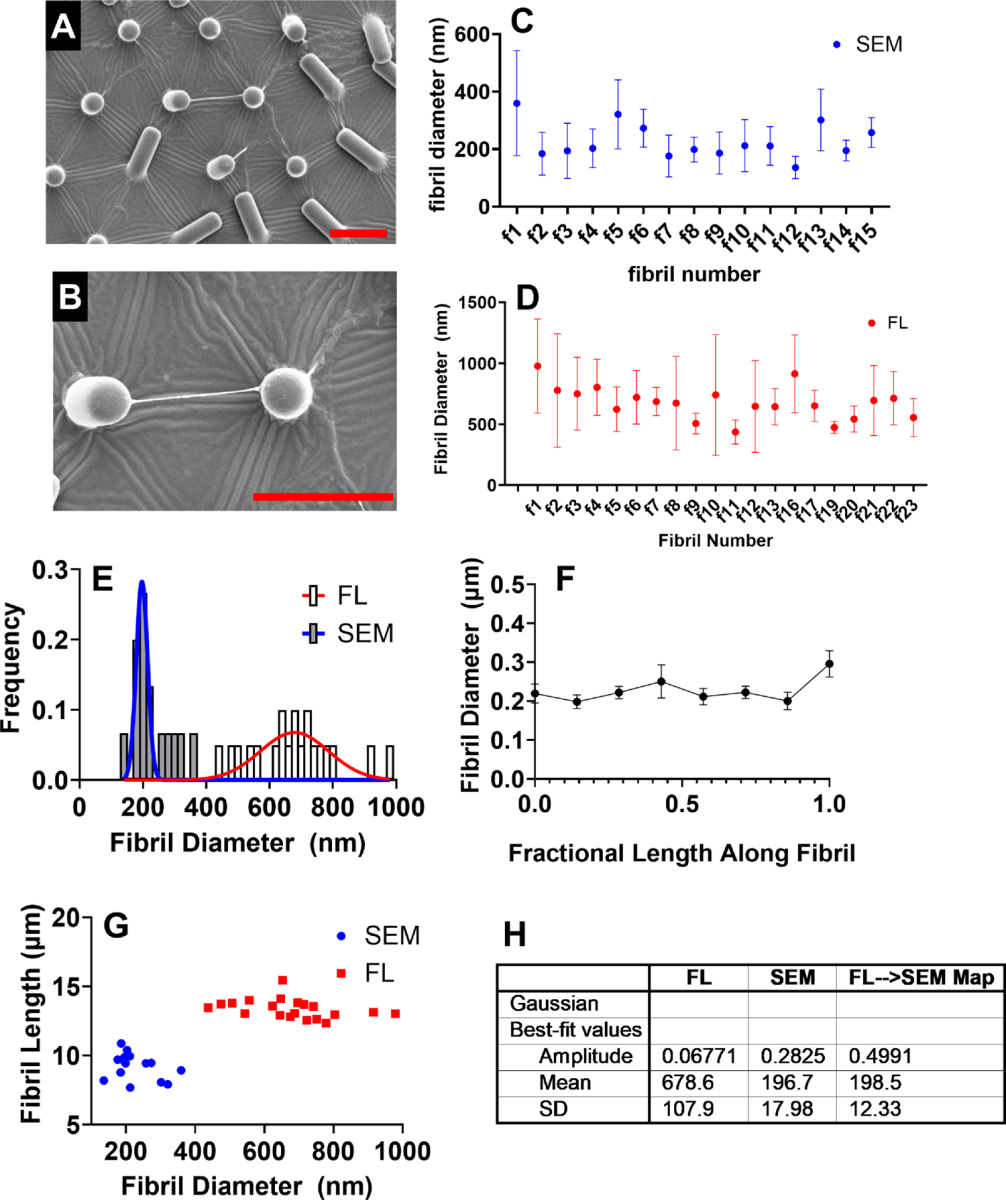
Quantifying FN fibril dimensions. (**A**–**B**) Scanning electron micrographs were acquired and used to calculate FN fibril diameter (scale bar = 10 µm). **C** Fibril diameter was measured at five (5) locations along each fibril (*N* = 15 fibrils), and the distribution of fibril diameters was compared to **D** the fibril diameter calculated from immunofluorescence images of fibrils (*N* = 20 fibrils). **E** Fibril diameter distributions were fit to a Gaussian equation. **F** Fibril diameter (as measured by SEM) as a function of normalized length along the fibril. **G** Fibril length versus fibril diameter indicates that fibril diameter is not a function of fibril length. **H** Gaussian mapping was used to calculate the fibril diameter from immunofluorescence images

Cell-derived FN fibrils have been previously shown to range from 5 to 1000 nm in diameter (Dzamba and Peters 1991) and up to 50 µm in length (Ohashi et al. 1999). One potential challenge of the current assay is that a diverse population of fibril diameters and lengths could confound mechanical testing results. A second challenge is that previous studies have indicated that the median FN fibril diameter is in the range of hundreds of nanometers (Dzamba and Peters 1991), which nears the resolution limit for fluorescence microscopy. To assess fibril diameter and lengths within a population of FN fibrils, we quantified FN fibril diameter and length via both immunofluorescence imaging and scanning electron microscopy. Figure 3A–B shows representative images of an FN fibril suspended between neighboring pillars. In order to limit variations in fibril length, we only examined fibrils that directly connect neighboring posts in both this analysis and the remainder of the current work. Note that critical point drying of microfabricated pillar arrays results in the collapse of many pillars but still allows for identification of a population of fibrils to measure. Figure 3C–D shows diameter measurements along fibrils using both scanning electron micrographs (SEM, Fig. 3C) and immunofluorescence images (FL, Fig. 3D). Unsurprisingly, fibril diameter measurements from immunofluorescence images were greater than those measured with SEM, given that SEM allows for better resolution of smaller structures, and given that fluorescence images were acquired using epifluorescence and a camera with a resolution of 160 nm/pixel. Figure 3E shows the histograms of fibril diameter for both measurement techniques, along with fitted Gaussian curves. Both techniques indicate a narrow deviation, suggesting less fibril diameter diversity than anticipated. Figure 3F shows fibril diameter as a function of length along the fibril, suggesting that diameters are roughly constant over the lenght of the fibril. Figure 3G indicates fibril length versus diameter, suggesting that there is no correlation between fibril diameter and length over the range of fibril lengths observed; this is an important observation, as it suggests that longer fibrils do not necessarily have corresponding increases in diameter, which would have implications for both stresses in a fibril and the elastic modulus of a fibril. (Note that differences in fibril length between SEM and immunofluorescence are most likely due to partial pillar collapse during critical point drying/SEM processing.) In order to determine a reasonable estimate of fibril diameter for the remainder of the current studies, we performed a Gaussian mapping of fibril diameters from immunofluorescence images onto SEM-measured diameters. This transformation was applied to fibril diameters and was used in subsequent mechanical testing experiments.

### Quantifying mechanical properties of FN fibrils

**Fig. 4:**
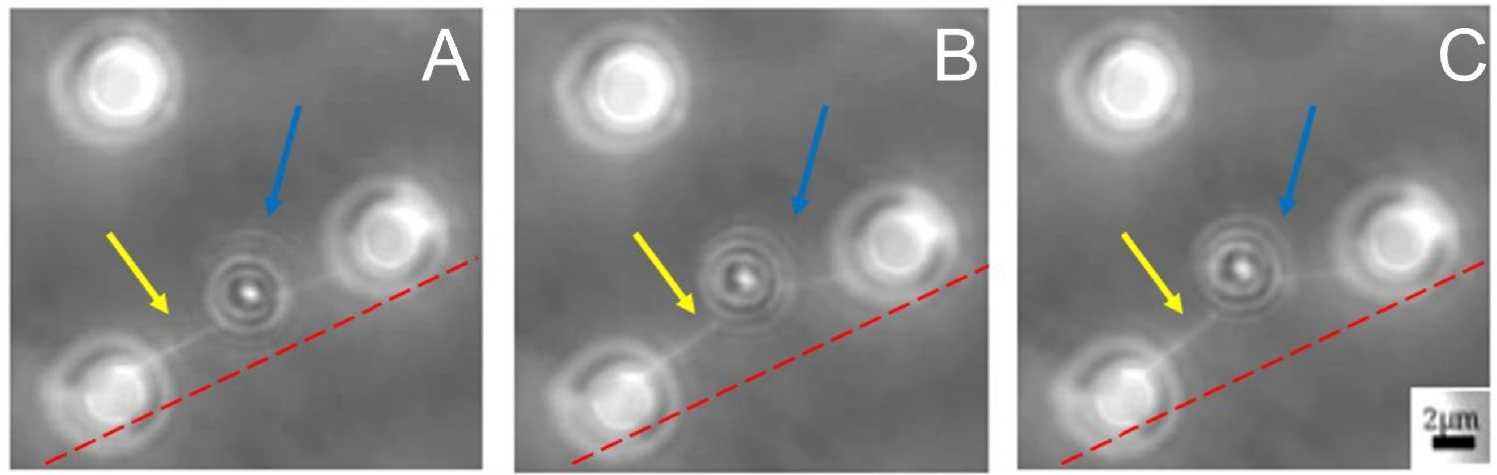
Optical tweezing of suspended FN fibrils. **A**–**C** Timelapse phase contrast images of a fibril (yellow arrow) being stretched by the bead (blue arrow) trapped in the optical tweezers. Red-dashed line is included as a reference position

Having established that isolated fibrils were primarily comprised of FN and having characterized fibril geometries, we next investigated whether optical tweezing of FN antibody-coated beads could sufficiently strain FN fibrils. A movie demonstrating the trapping process is shown in Supplemental Material Video S1. For uniformity of measurements, beads were placed as close to the center of each fibril as possible; distance of the bead along the fibril was measured and incorporated into the mechanical analysis discussed below. Figure 4A–C shows time lapse phase contrast images of a bead (blue arrow) being pulled in the optical trap and stretching the attached fibril (yellow arrow). The red line is added as a reference. A complete video of the stretching process is included in Supplemental Material Video S2. Supplemental Fig. S1 shows a fibril in which the resistive force exceeded the strength of the optical trap, resulting in the fibril-bead pair slipping from the trap to return to its original, straightened configuration and a loss of force. Note that as the trap is returned to its original position, the bead re-enters the trap, and the measured force returns to roughly the value observed before the bead left the trap. This suggests that the bead slipping out of the optical trap is the limiting factor in the system, and that fibril-bead rupture is not.

**Fig. 5:**
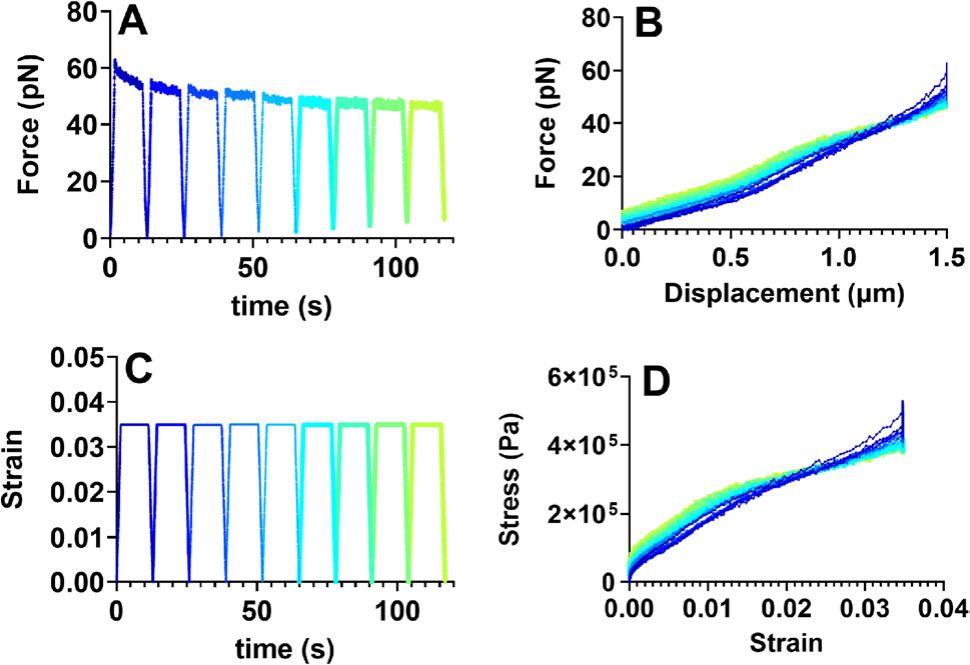
Elastic moduli of FN fibrils. Representative raw **A** force–time data and **B** force–displacement data for an FN fibril exposed to a repeated load-hold-unload strain pattern **C**. Raw data were transformed to **D** stress–strain data, as detailed in Supplemental Materials. Note that displacement in 5B refers to the bead displacement perpendicular to the fibril, whereas strain in C and D refers to the stretching of the fibril parallel to its axis. The transformations are described in Results and in more detail in SI Methods

In order to quantify both elastic responses and stress relaxation properties of fibrils, we subjected fibrils to the following strain regime: fibrils were stretched in a direction perpendicular to the fibril primary axis at a rate of 1 $\mu$m/s for 1.5 s (total displacement: 1.5 $\mu$m); held at 1.5 $\mu$m for 10 s to observe stress relaxation; and unloaded at the same 1 $\mu$m/s for 1.5 s back to the original position. This strain cycle was repeated nine (9) times per fibril (Fig. 5C), in order to quantify any pre-conditioning or cyclic responses. The maximum lateral displacement of 1.5 $\mu$m corresponds to a roughly 3% strain in the axial direction of the fibril. We intentionally limited the strain to this range in an attempt to avoid significant plastic deformation of the fibril. Note that the optical tweezers system has a linear range on the order of tens of microns; as such, all data collected was well within the linear range. Displacement and force data were acquired for each fibril, and a corresponding video was also recorded and used to measure fibril length, fibril diameter, and bead position along the fibril. Representative data for force versus time (Fig. 5A) and force versus displacement (Fig. 5B) curves are shown for multiple repetitions of the load-hold-unload test on a single FN fibril.

To determine the stress–strain relationship, several transformations of the data were performed. First, the geometry of the assay induces a lateral, “bowstring” displacement; this displacement was transformed to an axial extension using measured geometries of the fibril and bead position along the fibril. Axial extension was then transformed to engineering strain based on the fibril length. Force measurements were first transformed to 1st-Piola Kirchoff stresses and then to Cauchy stresses using the geometry of the fibril and an assumption of cylindrical shape (as described in Özkaya et al. (2016)). Full descriptions of these mathematical transformations are included in Supplemental Materials. Representative stress–time (Fig. 5C) and stress–strain (Fig. 5D) data are shown for a single fibril. Stress–strain curves and stress–time curves were calculated from raw data of 25 distinct FN fibrils.

Mechanical properties for the fibrils were next calculated from stress–strain and stress–time curves. Stress–strain curves during the load and unload phase of the applied strains was used to quantify elastic moduli, while stress–time curves during each of the hold phases was used to quantify stress relaxation properties. These data were used to determine the linearity of the elastic response as well as the viscoelastic properties of the fibrils.

#### Nonlinear elasticity of FN fibrils

**Fig. 6:**
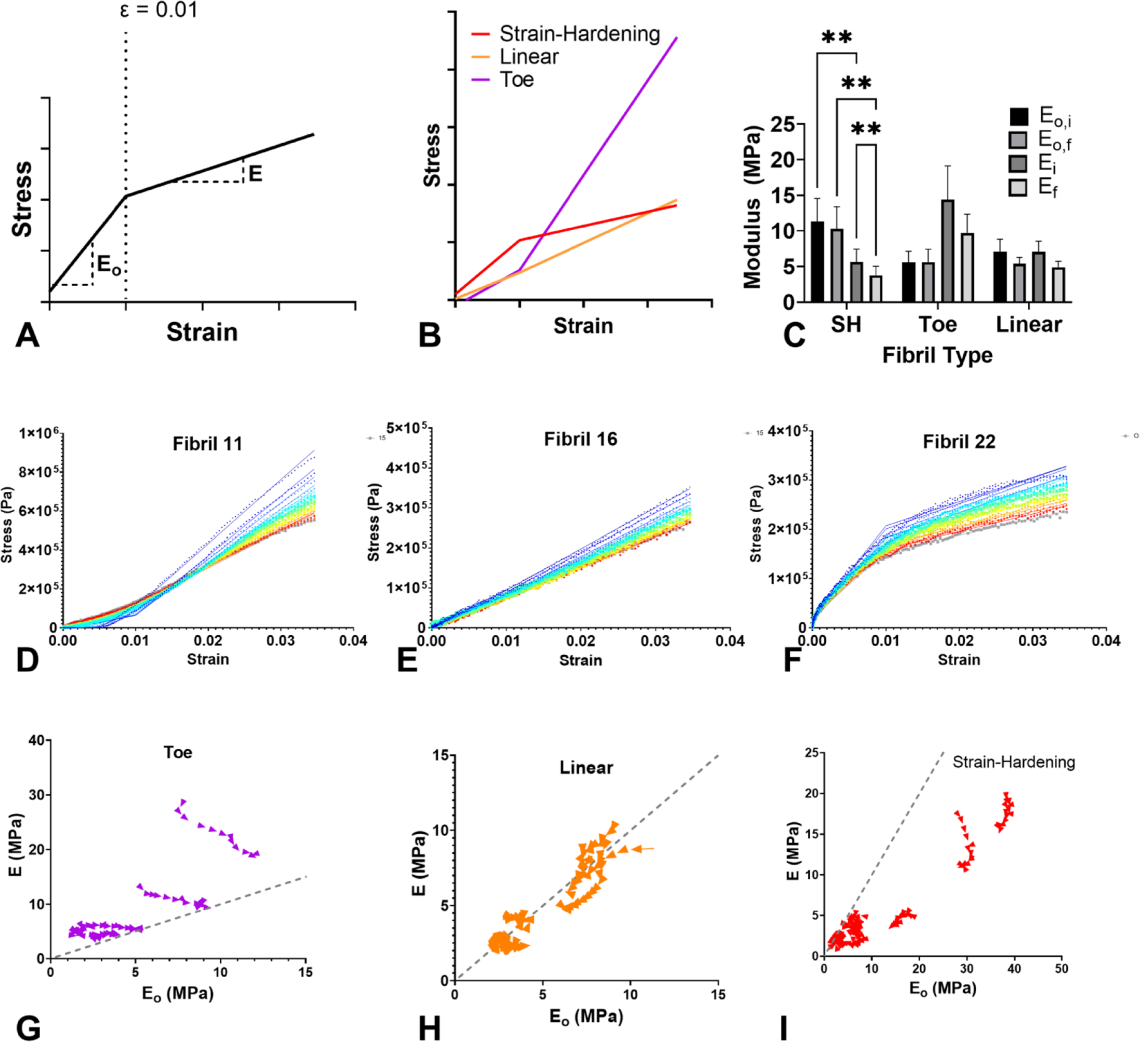
FN fibrils exhibit three distinct elastic responses. **A** The stress–strain curves for each fibril were fit to a piece-wise linear regression model, with a crossover point at $\epsilon$ = 0.01. Best-fit values were determined for slopes E_o_ and E. **B** Fibrils were classified into phenotypes shown based on relative values of E_o_ and E.**C** Plot for initial and final Eo and E values for each fibril phenotype. **D-F** Representative stress–strain curves for **D** a “toe” fibril, **E** a linear fibril, and **F** a strain-hardening fibril. **G-I** Changes in E_o_ and E as a function of successive stretches for **G** “toe” fibrils, **H** linear fibrils, and **I** strain-hardening fibrils. Arrows indicate direction from first to last stretch

The elastic response of fibrils was determined from the stress–strain curve of each fibril during the load phase of strain application (as stated above, fibrils were repeatedly loaded to a length of 1.5 $\mu$m at a rate of 1 $\mu$m/s). Each fibril was strained a minimum of nine (9) times, yielding nine distinct quantifications of the elastic response per each of the 25 fibrils. Complete stress–strain data for all 25 fibrils are shown in Supplemental Fig. 2. Initial observations indicated that fibril stress–strain responses could be fit with a piece-wise linear regression in which two distinct slopes are fit for strains $< 0.01$ and strains $> 0.01$. Fibrils for which an $R^2$ value greater than 0.9 (23 of the 25 fibrils) were used for subsequent analysis and classification. A graphical representation of this piece-wise linear fit is shown in Fig. 6A, where E_o_ and E represent the best-fit elastic moduli for strains $< 0.01$ and strains $> 0.01$ , respectively. Fibrils were classified based on the relative magnitude of E_o_ and E (Fig. 6B): fibrils in which E_o_ was < E are categorized as “toe”, as this shape is a classical biomechanical response of soft tissue described as having a toe region, originally by Fung (1996) (representative fibril shown in Fig. 6D, observed in 6 of 23 fibrils); fibrils in which E_o_ was $\approx$ E were classified as linear (representative fibril data as shown in Fig. 6E, observed in 5 of 23 fibrils); and fibrils in which E_o_ was > E are categorized as “strain-hardening”, as this shape is classically observed in many strain-hardening metals and has previously been shown to be fit well with a piece-wise linear model (originally described in Ramberg and Osgood (1943)) (representative fibril data shown in Fig. 6F, observed in 12 of 23 fibrils).

#### Pre-conditioning of FN fibrils

Many biological tissues exhibit “pre-conditioning”, in which the mechanical properties change over initial repeated stretching (Fung 1996). In order to quantify any pre-conditioning effects of strained fibrils, we quantified changes in both E_o_ and E over successive fibril loading. The change in stress–strain curves with repeated loading can be observed in the representative data as shown in Fig. 6D–F, with the initial loading colored blue and the final loading colored red. Changes in E_o_ and E are plotted for “toe” fibrils (Fig. 6G), linear fibrils (Fig. 6H), and strain-hardening fibrils (Fig. 6I). Several trends in elastic moduli can be observed: first, “toe” fibrils show an increase in E_o_ and a relatively constant E with successive loads. This indicates that these fibrils are approaching a linear elastic response, suggesting that the “toe” phenotype may be lost with pre-conditioning. Linear fibrils remain linearly elastic with successive pulls, with a slight decrease in the elastic modulus. “Strain-hardening” fibrils show a decrease in E and a relatively constant E_o_, indicating that the strain-hardening response becomes more pronounced with repeated strains, suggesting a possible exhaustion of the fibril. Table 1 summarizes average initial and final values of E_o_ and E (represented by “i" and “f" subscripts, respectively) for each fibril phenotype as well as for the entire population of fibrils. These values are also shown graphically in Fig. 6C.

**Table 1:** Piece-wise linear elastic moduli

Fibril classification	# of Fibrils	$\hbox {E}_{o,i}$ (MPa)	$\hbox {E}_{o,f}$ (MPa)	$\hbox {E}_{i}$ (MPa)	$\hbox {E}_{f}$ (MPa)
Toe	6	5.6 ± 1.6	5.6 ± 1.8	14.4 ± 4.7	9.7 ± 2.6
Strain-Hardening	12	11.3 ± 3.2	10.3 ± 3.1	5.7 ± 1.8	3.8 ± 1.3
Linear	5	7.1 ± 1.7	5.4 ± 0.9	7.1 ± 1.5	4.9 ± 0.8
All	23	8.9 ± 1.8	8.0 ± 1.7	8.3 ± 1.7	5.6 ± 1.1

Curve-fitting results for the piece-wise linear elastic constants (see Fig. 6A for graphical description). Results shown are mean ± SEM

#### Viscoelastic properties of FN fibrils

**Fig. 7:**
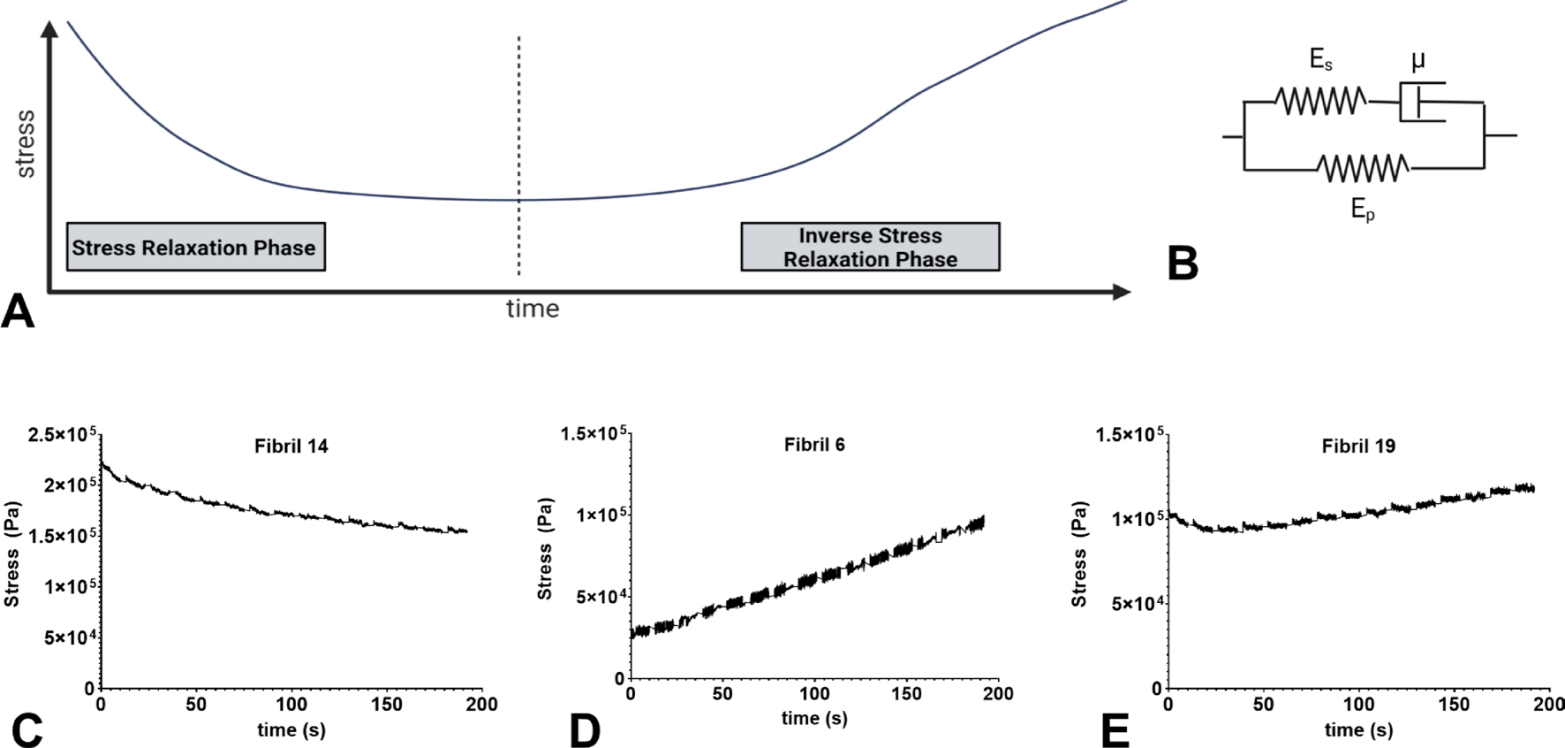
FN fibrils exhibit two distinct stress relaxation phases. **A** A schematic description of the stress relaxation and inverse stress relaxation phases. **B** The Maxwell standard linear solid viscoelastic model, in which a linear spring (elastic arm) is in parallel with a spring-dashpot combination (viscous arm). **C**–**E** Representative stress–time curves for **C** a fibril exhibiting stress relaxation, **D** a fibril exhibiting inverse stress relaxation, and **E** a fibril exhibiting both stress relaxation and subsequent inverse stress relaxation

We next quantified time-dependent mechanical properties of FN fibrils. While purely elastic materials do not relax under constant strain, viscoelastic materials exhibit a time-dependent stress relaxation (Fung 1996). Stress–time data for each fibril were compiled for the nine successive holds; data from all fibrils are shown in Supplemental Fig. S3. Initial observations indicated two distinct responses (shown schematically in Fig. 7A): first, a classic “stress relaxation”, in which stress falls asymptotically during constant strain (representative fibril data shown in Fig. 7C); and second, an “inverse stress relaxation”, in which stress increases during constant strain (representative fibril data shown in Fig. 7D). Note that this response is not commonly observed in biological tissues but has been previously observed in textiles (Vitkauskas 1998), silk (Kothari et al. 2001; Das and Ghosh 2006), and carbon nanotube yarns (Misak et al. 2013). Several fibrils exhibited both a stress relaxation phase and a subsequent inverse stress relaxation phase (representative fibril data shown in Fig. 7E), suggesting that the viscoelastic properties of FN fibrils may change over time, thus serving as a “mechanical memory” of fibrils. Stress–time data for each fibril were fit to the Maxwell standard linear solid (SLS) model of viscoelastic materials (Fig. 7B) (discussed in Fung (1996)). Table 2 shows the average values for E_p_, E_s_, and µ for both the stress relaxation and inverse stress relaxation phases. Note that since some fibrils exhibited both phases, the total number of fibril points in the analysis (30) exceeds the original population of fibrils considered (25). Additionally, it should be noted that two of the 25 fibrils showed a purely elastic response, in which no time-dependent changes were observed in stress. Best-fit values for E_s_ and µ are negative, which indicates the stress increase in this phase. The time constant for relaxation or inverse relaxation (defined as the ratio of µ to E_s_) was 49.3 s for the stress relaxation phase, and 313599 s (87.1 hr) for the inverse stress relaxation phase, suggesting that this phase may be the predominant phase in fibril mechanics.

**Table 2:** Viscoelastic constants

Relaxation phase	# of fibrils	$\hbox {E}_{p}$ (MPa)	$\hbox {E}_{s}$ (MPa)	$\mu$ (MPa/s)	$\tau$ (s)
Stress relax	17	8.6 ± 2.0	2.3 ± 0.6	115.2 ± 36.8	49.3 ± 16.3
Inverse stress relax	13	2772 ± 1362	−2760 ± 1362	−4.0e9 ± 0.4e9	313599 ± 22091

Curve-fitting results for the standard linear solid (SLS) viscoelastic model constants (see Fig. 7B for graphical description). Note that some fibrils exhibited both SR and IR phases, and as such the total number of data points is greater than the total number of fibrils. Results shown are mean ± SEM

## Discussion

FN fibrils play a critical role in embryogenesis, wound healing, and a host of pathologies. These fibrils are assembled via cell-derived contractile forces and served as a link between cells and their surroundings. FN fibrils serve as a provisional primordial matrix, which is subsequently remodeled as tissues mature and/or heal. Given that research over the past several decades has elucidated a significant role of mechanosensing in cellular signaling, the mechanical properties of FN fibrils are highly relevant to understanding signaling events in processes in which FN fibrils are involved. One of the significant limitations to measuring FN fibril mechanics is that FN fibrils are not easily replicated in cell-free environments. FN fibrils do not spontaneously assemble as collagen fibers and F-actin filaments do. While others have developed FN fibril-like structures, these do not replicate the morphology and deformability of cell-derived fibrils. We present here an assay that allows us to strain individual, cell-derived FN fibrils, and measure the responsive force. We have demonstrated that these fibrils remain intact after cell removal and that fibrils at 24 h are not decorated with several of the key ECM proteins that bind to mature fibrils.

**Fig. 8:**
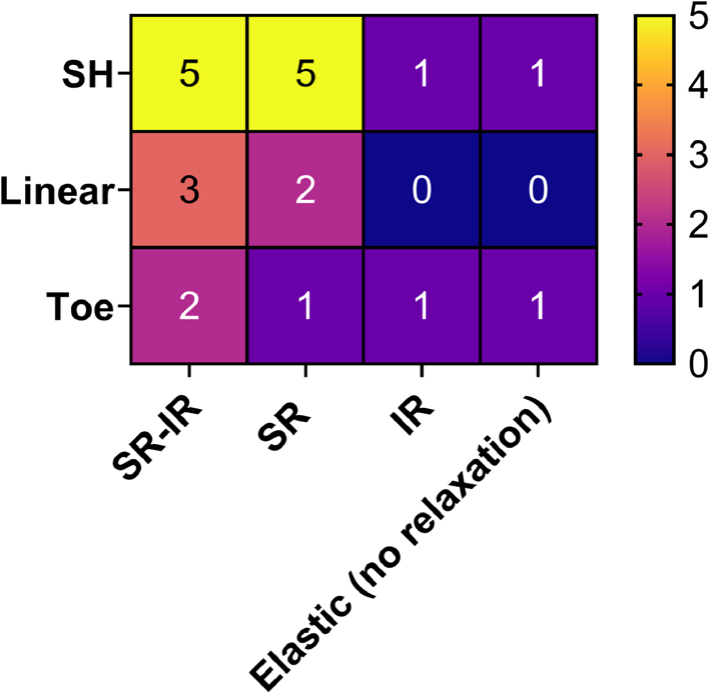
Frequency and correlation of elastic phenotypes and stress relaxation responses exhibited in FN fibrils. The number of fibrils exhibiting strain-hardening (SH), linear or toe elastic responses versus fibrils exhibiting stress relaxation (SR), and inverse stress relaxation (IR) or both (SR-IR)

Characterization of FN fibril mechanical properties revealed several unique features. First, fibrils exhibited three phenotypes of elastic response: (i) A “toe” phenotype, with a low elastic modulus at strains below 1% and a higher modulus above 1%; these fibrils showed a pre-conditioning response in which the elastic behavior became more linear with repeated stretching; (ii) a linear phenotype, in which the elastic modulus decreased with repeated stretching; and (iii) a “strain-hardening” phenotype, with a high elastic modulus at strains below 1% and a lower modulus above 1%; these fibrils showed a pre-conditioning response in which the strain-hardening became more pronounced with repeated stretching (that is, the elastic modulus for strains above 1% decreased with successive stretching). The entire population of fibrils exhibited a mean initial elastic modulus of approximately 9 MPa which decreased to approximately 8 MPa after repeated stretching. Characterization of the viscoelastic properties of FN fibrils indicated two distinct responses to constant strain: (i) a stress relaxation phase, in which stress decayed exponentially with time; and (ii) an inverse stress relaxation phase in which stress increased with time. Several fibrils exhibited both phases, suggesting that fibrils may transition from a stress relaxation response to an inverse stress relaxation phase. Curve-fitting the stress–time response to the Maxwell SLS model of viscoelasticity revealed a time constant $\tau$ of approximately 50 s for the stress relaxation phase versus a $\tau$ of approximately 81 h for the inverse stress relaxation phase, indicating that this response is most likely the predominant response experienced by fibril-attached cells. A summary of elastic and viscoelastic phenotypes is shown in Fig. 8. This summary indicates that the majority of fibrils exhibit a strain-hardening response, and a majority of fibrils exhibit both an initial stress relaxation response and a subsequent inverse stress relaxation response.

These findings have several critical implications, particularly in the fields of fibrotic disease and mechanotransduction. Extensive studies of mechanobiology have demonstrated that cells in stiffer environments exhibit increased contractile forces (Scott et al. 2015; Yang et al. 2011), decreased apoptosis (Leight et al. 2012), and increased differentiation into myofibroblasts (Olsen et al. 2011; Hinz 2010; Water et al. 2013), all of which promote FN assembly. Our results demonstrate that FN fibrils are stiffer than native liver (Yeh et al. 2002), lung (Lai-Fook and Hyatt 2000), kidney (Johnson et al. 2021), pancreas (Sugimoto et al. 2014), spleen, or intestinal tissue (Johnson et al. 2020). As such, FN fibril assembly in response to inflammation and/or repair mechanisms could induce a local increase in stiffness sensed by cells, which in turn could facilitate cellular responses that promotes further FN assembly. This unchecked positive feedback loop could promote fibrotic scarring due to mechanotransduction changes; indeed, inhibition of FN assembly has been shown to reduce liver fibrosis (Altrock et al. 2015) and kidney injury (Bowers et al. 2019), decrease contractile forces (Scott et al. 2015), and inhibit epithelial-mesenchymal transition (Griggs et al. 2017), which is a key cellular differentiation process implicated in fibrosis. Furthermore, our data suggest that FN fibrils store “mechanical memory"; that is, their stress is dependent upon previous deformation history. This suggests that mechanical properties of fibrotic tissue may change with fibril age, as has previously been suggested (Antia et al. 2008). The mechanism of a fast stress relaxation phase followed by a subsequent longer inverse stress relaxation phase may be explained by our knowledge of FN fibrillar structure: FN is known to exist in both a compact and extended conformation (Johnson et al. 1999); it is possible that the stress relaxation phase corresponds with the transition between these states in an FN fibril, resulting in stress relaxation. Once assembled, neighboring Type III domains in FN may bind to each other via beta-strand addition; this is a common binding mechanism in fibrillar and plaque proteins (reviewed in Richardson and Richardson (2002)). This cross-linking may serve to increase the stiffness of the fibril, resulting in an increased stress in response to constant strain.

There are some limitations of the current work. First, we have limited the pool of fibrils examined in terms of both assembly time and fibril size. Our data indicate that at later time points, fibrils are decorated with other ECM proteins, as would occur during tissue development and maturity. It is unclear whether these proteins alter the mechanics of the FN fibrils and/or whether the mechanics depend on the time that cells are given to assembly fibrils. The length of fibrils is nominally driven by the center-to-center distance of the microfabricated pillar arrays. Fibrils that span non-nearest neighbor pillars have been observed, but the effects of fibril length on fibril mechanics have yet to be systematically investigated. Second, removal of cells from the attached fibrils requires a buffer with detergent (0.5% Triton); it is possible that the process of cell removal alters fibril mechanics, although this concentration of Triton has previously been used to extract cells from fibronectin matrix and left a functional ECM (Griggs et al. 2017; O’Keefe et al. 1984). Third, the calculation of stress in the fibril is highly dependent on the measured fibril diameter. We have used diameters measured via SEM as has previously been done (Chen et al. 1978; Singer 1979; Minier et al. 1981); however, these may be affected by processing steps for SEM, and as such may represent a minimum diameter (and as such, a maximum stress). Future analysis of fibril diameters via high-resolution microscopy techniques will be conducted to further explore this concern. Finally, we have presented data only from adipose-derived mesenchymal stem cells. While we have previously demonstrated that other cell types can assemble FN fibrils that span micropillars (Lemmon et al. 2009), we have not systematically investigated whether fibril mechanics are cell-type specific. It would be of great interest to determine if fibrils from different cell types are capable of assembling fibrils with distinct mechanical properties.

## Conclusion

In conclusion, FN fibrils represent a key extracellular structure that is prevalent in a range of pathologies, in wound healing, and in embryonic development. Future work could provide substantial insight into the mechanics of a key component of the extracellular matrix. The assay presented here allows us to probe whether fibril mechanics change in longer fibrils; in more complicated fibril geometries, such as crosslinked fibrils; when assembled by different cell types and/or disease states; or when bound to other proteins. Given the prominent role of FN fibrils in the provisional ECM during wound healing and embryogenesis, these data would provide great insight into how mechanics of the ECM are altered across a host of physiological and pathological settings.

## Methods

### Cell culture

ASC52telo human adipose-derived mesenchymal stem cells (ATCCs) were grown at ${37}^\circ \hbox {C}$ and 5% CO2 in low glucose Dulbecco’s Modified Eagle’s Medium (Thermo Fisher, Bridgewater, NJ) supplemented with 2 mM L-glutamine (Sigma-Aldrich, St Louis MO), 10% fetal calf serum (Sigma-Aldrich, St Louis MO), 1% antibiotic: antimycotic (GeminiBio, West Sacramento, CA), and 1 ng/mL recombinant human basic fibroblast growth factor (Peprotech, Cranbury, NJ). Cells were maintained for less than 30 passages.

### Gel electrophoresis

100 uL of 200 nM FN was thawed to room temperature for 15 min and incubated with either trypsin or cell extraction buffer. Trypsin Inhibitor from Glycine max (10 mg/mL) (Sigma-Aldrich, St Louis MO) was added or an equivalent amount of Ca/Mg-free phosphate-buffered solution (PBS) (Quality Biological, Gaithersburg, MD), and samples in 1:1 Laemelli buffer were immediately prepared for gel electrophoresis (PowerPac Basic Mini, BIO-RAD, Hercules, CA) by an ${85}^\circ \hbox {C}$ water bath for 5 min and let cool; electrolysis ran for 29 min at 200 V, 40 A (constant) in 30 uL mini-PROTEAN TGX gels (4–20%, BIO-RAD, Hercules, CA).

### Micropillar array fabrication

Micropillar arrays (MPAs) were prepared as previous described (Scott et al. 2015). In brief, 2D array post patterns were created on 5 × 5 low-reflective chrome soda lime glass masks (Nanofilm, Westlake Village, CA) using a direct-write laser mask writer (microPatternGenerator 101, Heidelberg Instruments, Heidelberg, Germany). Masks were used to generate 10-micron tall features on SU-8 2010 spin-coated 4 inch silicon wafers (University Wafer, South Boston, MA) through UV exposure with a mask aligner (MA56, Karl Suss, Garching, Germany). Wafers were developed and submerged in organic solvents to prevent collapsing of high aspect ratio features before drying. These were baked at ${200}^\circ \hbox {C}$ for 1 h, cracked and incubated overnight in trichloro(1 H,1 H,2 H,2 H-perfluorooctyl)silane (TCP) vapor (Sigma-Aldrich, St Louis, MO) under 10-min desiccation after being exposed to oxygen plasma for 2 min (PDC-001-HP, Harrick Plasma, Ithaca, NY) to be used to generate negative stamps.

Negative stamps were cast by pouring 10:1 ratio of desiccated Sylgard 184 (DOWSIL, Midland, MI) polydimethylsiloxane (PDMS) elastomer base to curing agent overtop the silicon wafer in aluminum dishes, cooked at ${110}^\circ \hbox {C}$ for 10 min until the aluminum dish and wafer were cut free, and left to cure overnight at ${110}^\circ \hbox {C}$. Smaller squares outlining each post pattern were cut, exposed to oxygen plasma for 2 min (PDC-001-HP, Harrick Plasma, Ithaca, NY) and then incubated with TCP vapor overnight. These were used to generate positive post features by stamping them onto prepared PDMS droplets on clean 25 mm coverslips (cs) or 24x60 # 1 coverslides (CS) and cured overnight at ${110}^\circ \hbox {C}$. Negatives were cut free and peeled way to be reused until continuous predictable pattern collapse upon peeling and were recast.

### MPA preparation

Excess PDMS was removed from the positive post features on cs/CS and exposed to UV light for 10 min (PSD Pro Scan, Novascan, Chicago, IL). 100 uL of 200 nm FN was microcontact printed onto the tops of the activated posts after 30-min incubation on a 30:1 PDMS cube. cs/CS was submerged in 100% ethanol, 70% ethanol, subjected to three PBS washes and submerged in 3% F-127 Pluronic in PBS for 30 min to prevent cell attachment to the sides of posts. After another set of PBS washes, the prepared samples were stored in PBS until use. For some experiments, a 30-min 488- or 647-conjugated bovine serum albumin (BSA) (Invitrogen, Washington, DC) incubation was added before the F-127 step to label the pillars themselves, allowing for top and bottom imaging in force calculations by post deflection as previous reported (Scott et al. 2015).

Experiments carried out with cs were performed by placing the sample in a 6-well plate in 3 mL of media; those with CS were carried out in PDMS cell seeding molds: aluminum dishes were filled with 10:1 PDMS and fully cured. Octagons were cut out with square centers removed to be overlain onto the square post pattern on CS by applying a thin layer of vacuum grease (Dow Corning, Carrollton, KY) and filled to 0.5 mL. These were subsequently removed from CS after experimentation by slowly scraping in between with a thin tweezer and gradually lifting upward until completely free. Excess grease was removed from the molds and was cleaned by an ethanol submersion, dried to be reused.

### Isolation of fibronectin fibrils

Cells were removed via extraction buffer as previously done (Griggs et al. 2017) with subsequent PBS washes. For early time points, this was a 10-min incubation at ${37}^\circ \hbox {C}$; for longer time points, 20-min incubations were used at the same temperature. Extraction buffer was prepared as 20 mM ammonia hydroxide and 0.5% v/v Triton X-100 in PBS; double strength extraction buffer was prepared as 40 mM ammonia hydroxide and 0.1% v/v Triton X-100; ten-fold extraction buffer was prepared as 200 mM ammonia hydroxide and 1.0% v/v Triton X-100.

### Immunofluorescence imaging

In non-decellularized MPA samples, cells were fixed and permeabilized with 4% paraformaldehyde and 0.5% v/v Triton X-100 for 2 min, rinsed and incubated in 4% paraformaldehyde for 20 min; decellularized MPAs were instead incubated in extraction buffers. All samples were stained with one or more of the following: AlexaFluor555 phalloidin (Invitrogen, Washington, DC) to label F-actin, DAPI (Sigma-Aldrich, St Louis, MO) to label cell nuclei or DNA debris, polyclonal anti-FN antibodies (Abcam, Waltham, MA) conjugated to one of AlexaFluor647 goat anti-chicken secondary (Life Technologies, Eugene, OR), AlexaFluor647 goat anti-rabbit secondary (Life Technologies, Eugene, OR), or AlexaFluor488 donkey anti-rabbit secondary (Life Technologies, Eugene, OR), or Protein G coated polystyrene particles (Spherotech, Libertyville, IL) conjugated with polyclonal anti-FN antibodies and AlexaFluor488 goat anti-rabbit secondary; for ECM staining experiments, rabbit polyclonal collagen V (Abcam, Waltham, MA), rabbit polyclonal collagen I (Abcam, Waltham, MA), or mouse monoclonal elastin (Millipore Sigma, St Louis, MO) antibodies conjugated to the correct species and appropriately available channel with respect to FN staining options were utilized. CS were mounted to glass slides with fluoromount-$\hbox {G}^{\text {TM}}$ or vacuum grease; CS were mounted to glass slides with vacuum grease and pressed to approximately 1.27mm using a Mitutoyo digimatic micrometer MDC-SX. Immunofluorescent images were taken on a Zeiss Axiovert inverted fluorescence microscope equipped with a CCD camera (Zeiss, Oberkochen, Germany) and processed through ZEN software (Zeiss, Oberkochen, Germany).

### Optical tweezing

Optical tweezing was carried out using the dual-beam optical tweezers setup $\hbox {NanoTracker}^{\text {TM}}$ 2 v3.0 (JPK Instruments, Berlin, Germany) assembled on a Nikon Eclipse Ti Inverted Optical Microscope platform (Nikon, Tokyo, Japan) as has previously been used to quantify forces on DNA (Gibbs et al. 2021). The system was modified for the current study as follows: a 3W-, 1064-nm continuous wave laser was used to create the optical traps and was calibrated for the trap stiffness using the JPK built-in single-button calibration procedure while the displacement of the trap was monitored by a quadrant photodiode (QPD). The detection objective is a CFI Apochromat 60x/1.0 W, WD 2.8 mm, NA 1.0 while the trapping objective is a CFI Plan Apochromat “VC” 60x WI, WD 0.28 mm, NA 1.2, and the instrument is capable for fluorescence visualization via an epifluorescence light source (PhotoFluor II 89 North with liquid light guide) with a filter set available (450-490/505/520). The sampling rate of this instrument is 800 kHz, which allows less than 2 $\upmu$s time resolution; similarly, the sensitivity for force measurement is down to 0.1 pN, recording force fluctuations with high resolution. The combination of which provides precise trapping of single specimen with a high spatiotemporal resolution. For the current experiments, a custom flow cell containing MPAs with cell-derived fibrils was assembled into a glass slide “sandwich”. Unlike dueal-trap experiments for DNA (Gibbs et al. 2021), a single trap was used for fibril stretching.

### Data processing

Data were collected and rendered into.txt formats using the NanoTrackerTM Data Processing Software v2.5 (JPK Instruments, Berlin, Germany). These files were imported into Microsoft Excel and formatted into cells specified by user written Matlab code in concordance with tweezer testing type as set forth by the data output from the $\hbox {NanoTracker}^{\text {TM}}$. X and Y data were transformed into resultant forces through vector addition and multiplied into pico scale force ranges or into micron scale ranges for displacements. Loading and unloading data were processed separately but were subjected to similar analyses. Displacements were triangulated into extended fibrillar segments and summed to get a stretch ratio as a function of time.

## Supplementary information

Supplementary information and figures are included in an attached SI file.

## Supplementary Information

Below is the link to the electronic supplementary material.

Supplementary file 1 (pdf 826 KB)

Supplementary file 2 (mp4 622 KB)

Supplementary file 3 (mp4 1719 KB)

## Data Availability

All data files are stored in either.mat files (raw optical tweezer data) or GraphPad Prism.pzfx files (processed stress–time data and elastic moduli data). Upon publication, these data will be publicly available from the figshare.com database (DOI 10.6084/m9.figshare.23750745).
