# Supplementary material for: Measuring the biomechanical properties of cell-derived fibronectin fibrils
Source: Biomech Model Mechanobiol. 2024 Dec 26;24(2):455–69. doi: 10.1007/s10237-024-01918-3 (PMC12055646; doi:10.1007/s10237-024-01918-3)
Supplement: Supplementary file 1 — (pdf 826 KB) [file 10237_2024_1918_MOESM1_ESM.pdf]

Supplementary Materials for

Measuring the Biomechanical Properties of Cell-Derived Fibronectin Fibrils

Caleb J. Dalton *et al.*

*Corresponding author. Email: clemmon@vcu.edu

This PDF file includes:

Supplementary Text
Fig. S1 to S3
Movies S1 to S2

Other Supplementary Materials for this manuscript include the following:

Movies S1 to S2

Supplementary Text

Transformation of Force-Displacement Data to Stress-Strain Data

Raw extension and force data were transformed into stress-strain curves via the following approach. First, axial extension of the fibril was calculated from lateral extension data (i.e., the "bowstring" configuration) using the Pythagorean theorem, the bead position along the fibril, and the fibril length. Axial extension was converted to strain (ε) by the equation:

$$\varepsilon = \frac{L - L_0}{L_0} \qquad \text{(Eq. 1)}$$

where L is the deformed length of the fibril and L_0 is the initial length of the fibril. Stress was calculated by treating FN fibrils as cylindrical beams with simply supported ends and loaded at an arbitrary distance along the fibril corresponding to the location of bead attachment. While this is rotated 90 degrees from the classic beam-bending geometry, the math is no different than classical mechanics problems of a beam subjected to a load at a point along the beam. Regarding the cross-sectional geometry of the beam, while previous modeling efforts have assumed hexagonal packing within an FN fibril (*1*, *2*), the cross-sectional geometry of a fibril has not been explicitly explored, so a circular cross-section was assumed.

The FN fibril was modeled in cylindrical coordinates. All lateral forces within the fibril face would be supplied by FN-FN molecular bonds within the fibril, applied in the r and θ directions. However, as the fibril does not disintegrate during optical tweezing, we can eliminate internal mechanisms in favor of the overall mechanics of the motion: a plastic bead conjugated with antibodies with affinity for FN is electrostatically connected to the fibril and supplies sufficient force for perpendicular displacement, which, with its ends pinned, bends the fibril. Invoking incompressibility, the fibril is stretched, or axially strained, by a factor of λ, its cross-sectional area is reduced by a factor of 1/λ, and we neglect twisting and shear forces of molecular sliding. This gives the resulting deformation tensor in cylindrical coordinates:

$$\tilde{F} = \begin{bmatrix} \frac{1}{\lambda} & 0 & 0 \\ 0 & 1 & 0 \\ 0 & 0 & \lambda \end{bmatrix} \qquad \text{(Eq. 2)}$$

The only applied stress is the bending stress, Mc/I, which is a normal tensile force along the outer curve and compressive force along the inner curve. This stress is across the cross sectional face such that is in the z direction. This gives the resulting stress tensor in cylindrical coordinates:

$$\tilde{\Sigma} = \begin{bmatrix} 0 & 0 & 0 \\ 0 & 0 & 0 \\ 0 & 0 & \Sigma_{zz} \end{bmatrix} \qquad \text{(Eq. 3)}$$

The 1st Piola-Kirchhoff Stress, $\tilde{T} = \tilde{\Sigma}$, is defined as the measured stress, σ = f / A_0. The Cauchy (Eulerian) stress, $\tilde{\sigma} = \tilde{T}\lambda = \tilde{\Sigma} \bullet \tilde{F}$ is the engineering (true) stress, σ = f / A (see (*3,4*) for further study). With predominantly sparse matrices, this is reduced to scalar multiplication:

$$\sigma = \Sigma_{ZZ}\lambda \qquad \text{(Eq. 4)}$$

$$\sigma = \frac{\mathrm{McL}}{\mathrm{IL_0}} \qquad \text{(Eq. 5)}$$

$$\sigma = \frac{4\mathrm{abF}_{OT}}{\pi r^3 L_0} \qquad \text{(Eq. 6)}$$

where a and b are the distances from either end of the fibril to the point of bead attachment (such that a + b = L_o), F_{OT} is the force applied by the optical tweezers, and moment M = a*b*F_{OT}/L, as calculated for a perpendicular force applied at the center of a beam (4). Thus, stress can be calculated via Eq. 6 given the initial length of the fibril L_0, the force applied F_{OT}, the lengths from fibril end to the attached bead (a and b), and the radius of the fibril r.

1. C. A. Lemmon, S. H. Weinberg, Multiple Cryptic Binding Sites are Necessary for Robust Fibronectin Assembly: An in Silico Study. *Sci Rep*. **7** (2017), doi:10.1038/s41598-017-18328-4.
2. S. H. Weinberg, D. B. Mair, C. A. Lemmon, Mechanotransduction dynamics at the cell-matrix interface. *Biophys J*. **112**, 1962–1974 (2017).
3. N. Özkaya, D. Goldsheyder, M. Nordin, D. Leger, *Fundamentals of biomechanics: Equilibrium, motion, and deformation, fourth edition* (2016).
4. Fung, Y.C.: *Mechanical Properties of Living Tissues* (1996).

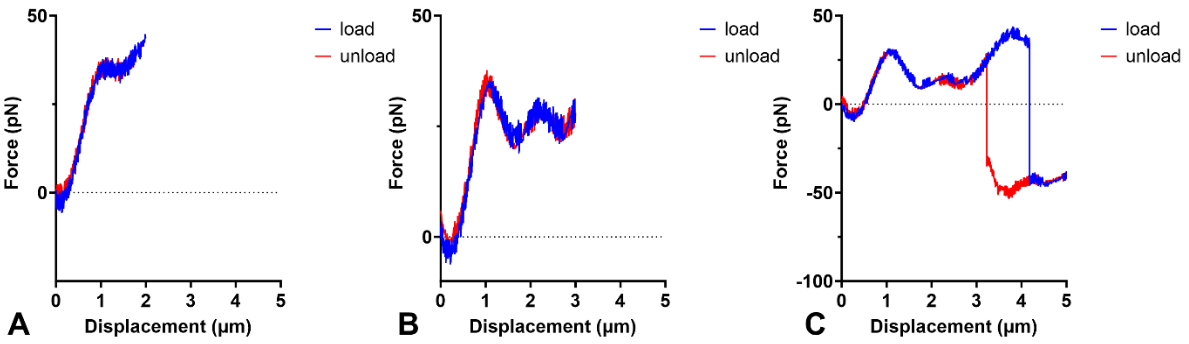

Fig. S1. Bead slippage from the trap is the limiting factor in optical tweezing of FN fibrils. To test the limits of optical tweezing of FN fibrils, fibrils were repeatedly stretched to increasing displacements. Data from a representative fibril is shown with successive stretches to (A) 2 µm, (B) 3 µm, and (C) 5 µm. Note that in (C), force data drops around 4 µm, but is recovered during the unloading phase at around 3 µm. This suggests that the bead slipped from the trap and stretching was recovered when the laser was returned to a position close enough to recapture the bead. If rupture of the fibril occurred, then there would be no signal recovery during unloading; if the bead ruptured from the fibril, there would be no signal recovery until the laser returned to the initial position and the bead was able to reattach to the fibril.

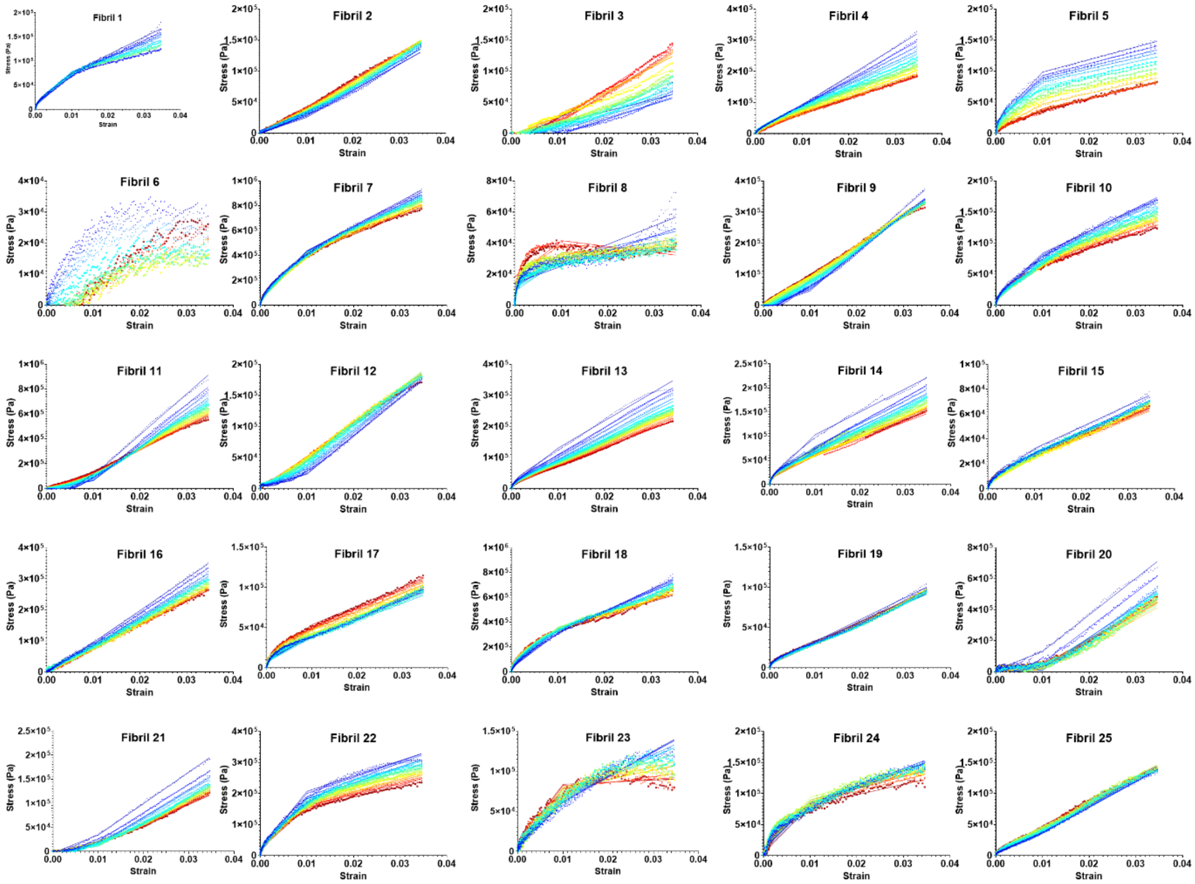

Fig. S2. Complete stress-strain dataset for all fibrils. Stress-strain curves for 25 fibrils. Each fibril was stretched 9 times; colors indicate progression from first stretch (blue) to last stretch (red).

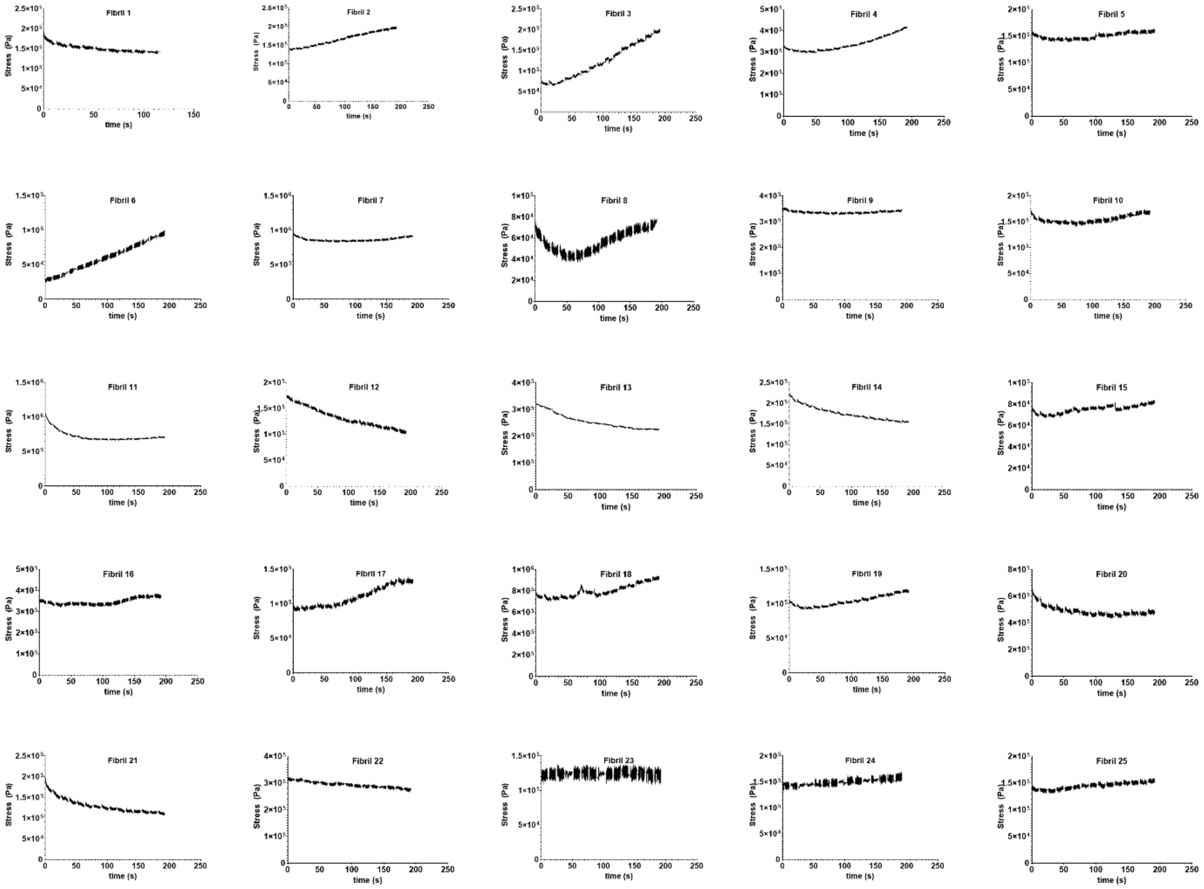

Fig. S3. Complete stress-time dataset for all fibrils. Stress-time curves for all fibrils subjected to constant strain.

Movie S1.

A fluorescence movie of a bead being trapped by the tweezers and moved into conformal contact with an FN fibril. Purple circle indicates initial position of bead.

Movie S2.

A fluorescence movie of an FN fibril being stretched by an attached polystyrene bead trapped in the optical tweezers. Red and blue lines are added for visual reference positions.
